# Invagination intestinale sur diverticule de Meckel chez l´adulte: à propos d´un cas

**DOI:** 10.11604/pamj.2021.39.86.27184

**Published:** 2021-05-28

**Authors:** Abdelali Guellil, Rachid Jabi, Laila Bouzayan, Mohammed Boudou, Soumia Elarabi, Brahim Zeriouh, Mohammed Bouziane

**Affiliations:** 1 Service de Chirurgie Viscérale et Oncologie Digestive A, CHU Mohammed VI, Oujda, Maroc

**Keywords:** Diverticule de Meckel, invagination intestinale, occlusion, adulte, chirurgie, Meckel´s diverticulum, intussusception, occlusion, adult, surgery, a case report

## Abstract

Le diverticule de Meckel reste l´anomalie congénitale la plus courante affectant le tractus gastro-intestinale, correspondant au défaut d´involution du canal omphalo-mésentérique. Cette anomalie est le plus souvent asymptomatique, et peut-être révélé lors d´une complication tel que l´invagination intestinale. Cet article présente le diagnostic et la prise en charge d´une patiente de 18 ans admise en urgence pour invagination iléo-iléale sur diverticule de Meckel compliqué d´occlusion. Le report de cette observation est intéressant, car elle met en lumière une pathologie rare dont le diagnostic doit être précoce par scanner abdominal, qui permet d´éviter la perforation ou la nécrose grêlique. La résection intestinale sans désinvagination est le traitement de référence.

## Abstract (en)

Meckel´s diverticulum is the most common congenital anomaly affecting the gastrointestinal tract resulting from failure of involution of the omphalomesenteric duct. This anomaly is most often asymptomatic and may be revealed by a complication, such as intussusception. This study reports the diagnostic pathway and the management of an 18-year-old female patient admitted to the Emergency Department with ileoileal invagination due to Meckel´s diverticulum complicated by occlusion. This study is interesting because it provides an overview of this rare disease whose diagnosis, based on abdominal scanner, must be made early in order to prevent small bowel perforation or necrosis. Bowel resection without desinvagination is the gold standard treatment.

## Introduction

Le diverticule de Meckel est l'anomalie congénitale la plus fréquente du système gastro-intestinal. Il résulte d'un canal vitellin incomplet. Retrouvé dans 0,14% à 4,5% des dissections de cadavres [1], avec une atteinte de 0,014% femme pour un homme et l´âge médian de 42 ans [1]. Dans la majorité des cas, il est asymptomatique. Comme il peut se compliquer d´une invagination intestinale avec occlusion grêlique. Ces cas peuvent être très dangereux s'ils ne sont pas détectés et peuvent évoluer vers une nécrose intestinale, une perforation et une septicémie [2]. Entités très rares et inhabituelles de l´adulte, de découverte fortuite devant un tableau aigu d´occlusion. L´objectif de cet article est de mettre le point sur l´invagination intestinale de l´adulte sur le diverticule de Meckel et de décrire les différentes modalités diagnostiques et thérapeutiques.

## Patient et observation

**Figure 1:**
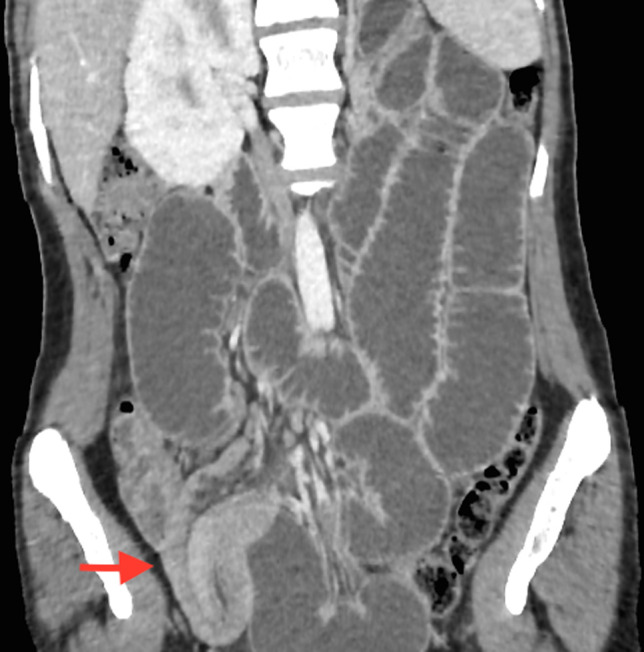
coupe scanographique coronale montrant l´aspect en sandwich

**Figure 2:**
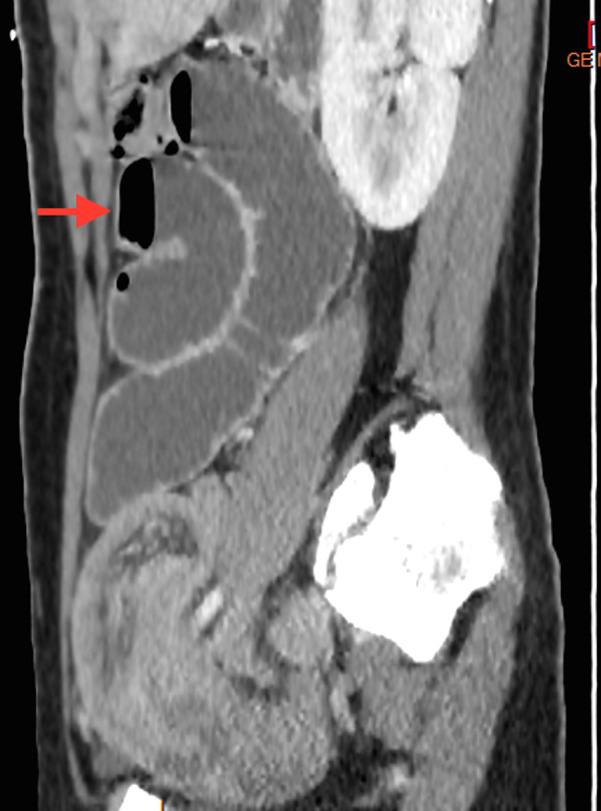
coupe sagittale montrant une importante distension des anses grêliques mesurant 35mm de diamètre maximal renferment des niveaux hydro-aérique

**Figure 3:**
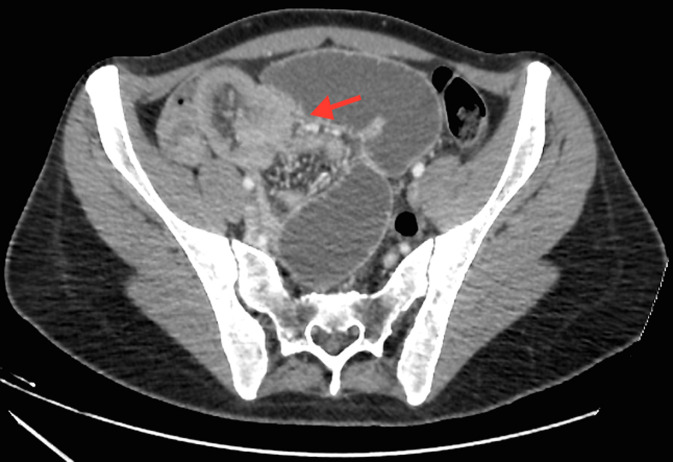
tomodensitométrie (TDM) abdominale en coupes axiales après injection de produit de contraste montrant l´aspect en cocarde rappelant l´aspect d´un boudin d´invagination grêlo-grêlique (iléo-iléale), étendu sur plus de 115mm, situé au niveau de la fosse iliaque droite et étendu au pelvis, sur un diverticule de Meckel

Nous rapportons l´observation d´une patiente âgée de 18 ans, sans antécédents, admise au service d´urgence pour syndromes occlusifs. À son arrivée, la patiente était apyrétique, stable sur le plan hémodynamique avec une tension artérielle à 140/70 mmHg, un bon état général. L´examen abdominal révélait une douleur abdominale diffuse avec notion d´arrêt des matières et des gaz et de vomissements bilieux avec un abdomen légèrement distendu. Le bilan biologique révèle un syndrome inflammatoire (CRP = 21 mg/L). La radiographie de l'abdomen sans préparation montrait des niveaux hydro-aériques grêliques. La tomodensitométrie abdominale réalisée en premier lieu, vue que la patiente était en occlusion, évoquait le diagnostic en montrant une occlusion grêlique sur invagination iléo-iléale sur un Diverticule de Meckel avec épaississement pariétal digestif de l´anse incarcérée (Figure 1, Figure 2, Figure 3).

**Figure 4:**
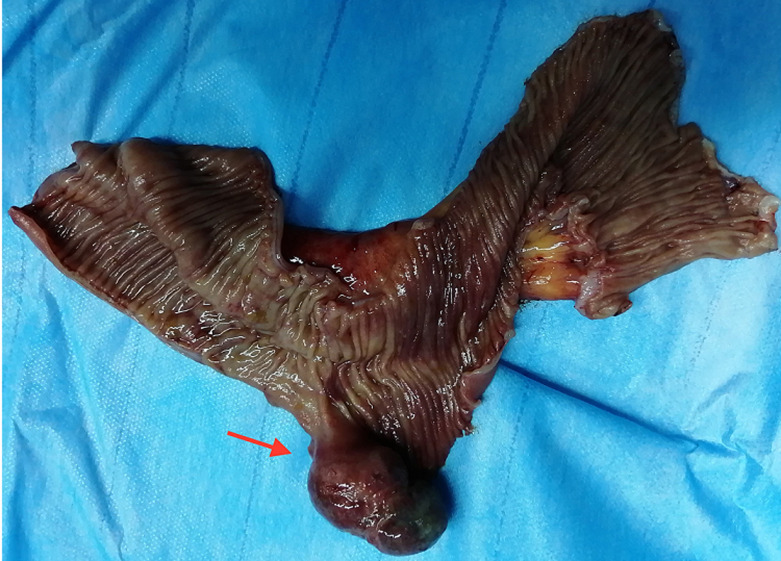
pièce opératoire de résection segmentaire montrant le diverticule de Meckel

**Figure 5:**
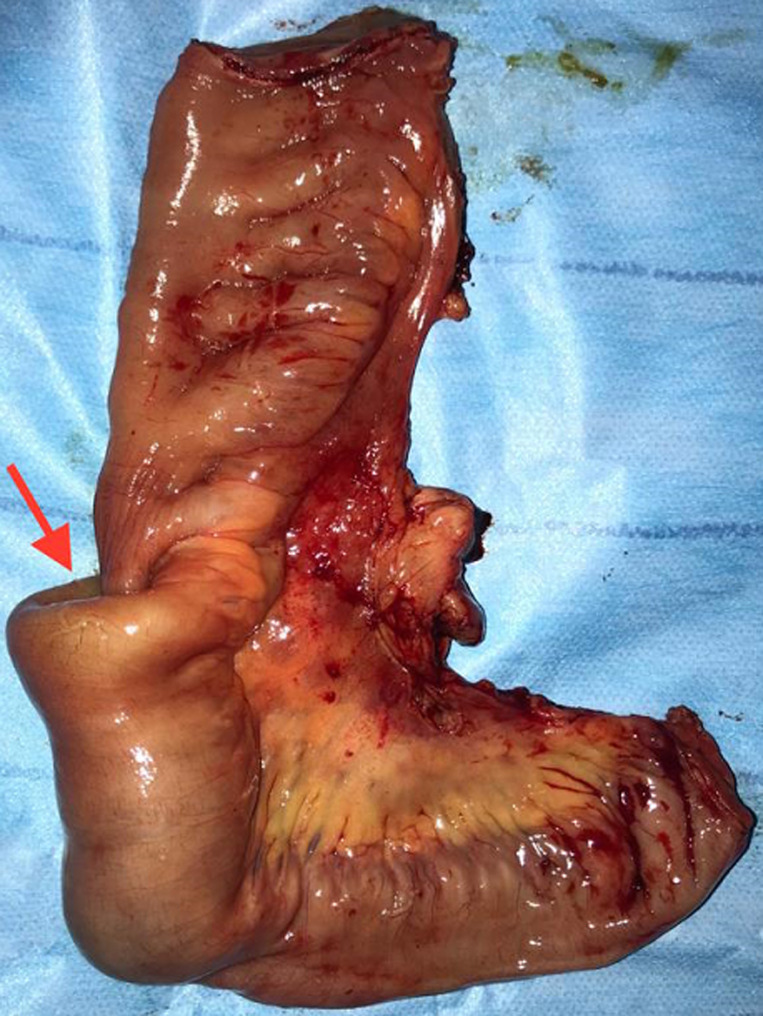
pièce opératoire montrant l´invagination iléo-iléale

L´indication chirurgicale se posait en urgence devant ce tableau clinique d´occlusion sur invagination. L´acte opératoire confirmait l´invagination iléo-iléal sur Diverticule de Meckel, situé à 1 mètre de la valve de Bauhin (Figure 4, Figure 5). Nous avons réalisé une résection grêlique emportant l´invagination et le Diverticule (15cm) avec anastomose grêlo-grêlique término-terminale manuelle. Les suites postopératoires immédiates ont été simples, autorisant la sortie au sixième jour postopératoire. L'examen anatomopathologique du diverticule a montré un tissu fibreux œdématié et lâche par endroit abritant un infiltrat inflammatoire polymorphe. La muqueuse était semblable à celle de la muqueuse gastrique.

## Discussion

Les complications du diverticule de Meckel sont rares, estimées à 4% sur la durée totale de la vie, maximale avant 2 ans, environ 1% vers 40 ans et quasi nulle après 70 ans [1]. L´âge moyen de survenue de complications est estimé à 2,8 ans [1], loin de celui rapporté par notre étude. On distingue les complications hémorragiques, mécaniques, infectieuses et tumorales.

L´occlusion révélatrice de 14 à 40% des Diverticules symptomatiques de l´adulte [1], peut avoir diverses causes, dont la principale est l´invagination sur Diverticule [1]. L´invagination intestinale aiguë a été décrite pour la première fois par Barbette en 1674 [3]. En 1871, Sir Jonathan Hutchinson fut le premier à opérer avec succès un enfant atteint d´invagination [4]. Parmi la littérature actuelle, un seul cas de Diverticule de Meckel mésentérique a entraîné une invagination [5] dans l´étude de McGrath *et al*. à Londres, et c´est le cas d´un patient contrairement à notre cas [5]. Sa physiopathologie n´est pas bien comprise, mais certains auteurs suggèrent que la présence de muqueuse gastrique peut entraîner un mouvement péristaltique anormal [6], ce qui est notre cas vue la présence de cette muqueuse. Le diagnostic de l´invagination intestinale a été posé après examen tomodensitométrique malgré l´importance d´échographie abdominale qui reste gênée par la présence d´air en cas d´occlusion. La même stratégie diagnostic a été adoptée chez McGrath *et al*. sauf que dans une série de Traoré *et al*. en Mali [7] à propos de 41 patients, l´échographie abdominale a objectivé le boudin d´invagination sur 11 cas.

Iléon est la localisation la plus fréquente de l´invagination intestinale, 27% de localisation colo-coliques, et rarement colorectales ou colo anales [8]. Ainsi que la localisation du Diverticule de Meckel varie entre 10 et 100 cm par rapport à la valvule de Bauhin dans 50% des cas. Ses dimensions sont en moyenne 2cm de diamètre, 5cm de longueur [9]. Chez notre patiente la localisation du boudin d´invagination se situe vers 115 mm du carrefour iléo-coecale, même localisation chez l´étude de McGrath *et al*.

La chirurgie reste le meilleur traitement de l´invagination chez l´adulte. La plupart des auteurs admettent la nécessité d´une laparotomie exploratrice. La réduction de l'invagination est déconseillée par la majorité des auteurs en cas de signes de souffrance intestinale et en absence de preuve préopératoire formelle de bénignité de la lésion causale [10]. Nous avons réalisé une résection intestinale par laparotomie avec anastomose iléo-iléale termino-terminale par défaut de laparoscopie en urgence. Contrairement à McGrath *et al*. qui ont abordé le malade par laparoscopie avec résection grêlique et anastomose iléo-iléal latérale. L´évolution post opératoire a été sans incident pour nos deux patients (notre patiente et le patient de McGrath *et al*).

## Conclusion

L´occlusion sur invagination ileo-ileale secondaire à un Diverticule de Meckel reste une entité diagnostiquée fortuitement ou lors d´apparition de complications telles que la nécrose, la perforation intestinale. On n´a pas de consensus sur la prise en charge du Diverticule de Meckel asymptomatique pour éviter tels complications. L´intervention de référence reste la résection segmentaire avec anastomose afin d´être certain de ne pas laisser en place de muqueuse ectopique.
